# In the Spotlight—Established Researcher

**DOI:** 10.1002/jez.b.23318

**Published:** 2025-07-24

**Authors:** Erich Bornberg‐Bauer

**Affiliations:** ^1^ Institute for Evolution and Biodiversity University of Münster Münster Germany; ^2^ Department Protein Evolution Max Planck Institute for Biology Tübingen Germany


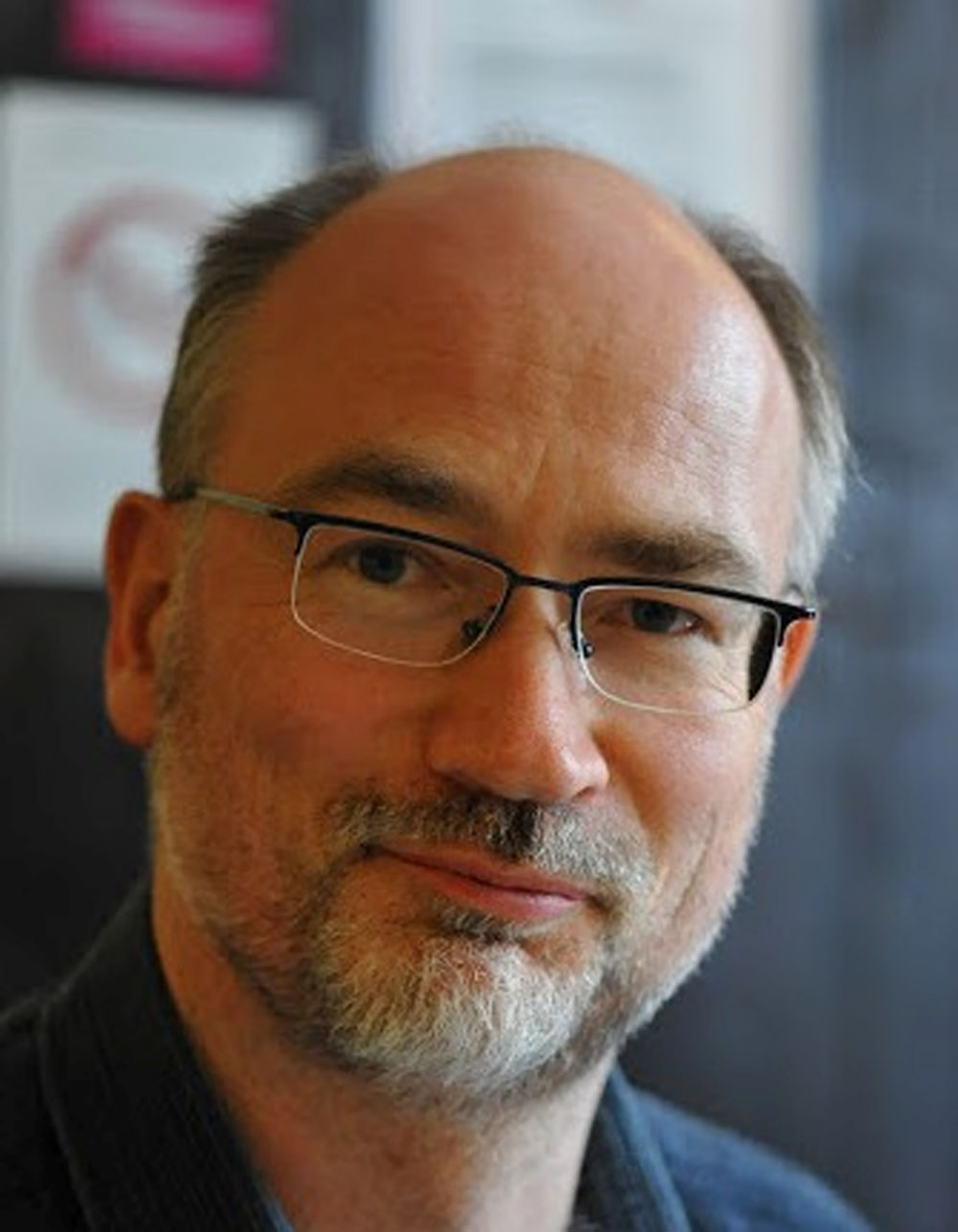


Professor Erich Bornberg‐Bauer is an internationally recognized expert in molecular evolution and bioinformatics, with a career that spans over three decades and bridges disciplines from biophysics to experimental genomics. Currently a full professor at the University of Münster, his work aims to shape our understanding of how new biological functions evolve, how novel genes arise, and how protein architecture and modularity influence organismal complexity.

Erich Bornberg‐Bauer is the Coordinator of the DFG Priority Programme “Genomic Basis of Evolutionary Innovations” (SPP 2349, GEvol) and a Principal Investigator in HFSP, DrosEU, EvoPAD, and multiple EU Horizon 2020 projects.

Erich Bornberg‐Bauer is a Guest Co‐Editor of this special issue on the *Genomic Basis of Evolutionary Innovations in Insect*s.

**Website**: https://bornberglab.org

**Google Scholar page**: https://scholar.google.de/citations?hl=en&user=cuyRZ88AAAAJ&view_op=list_works&pagesize=100@.

## 1 With Whom and Where Did You Study?

I studied Biochemistry and Mathematics at the University of Vienna, and initially got interested in theoretical biology, Turing models, and other models describing development in the first place. I completed my PhD with Peter Schuster, who trained us in computational molecular evolutionary biology and taught us to think in evolutionary terms. After postdoctoral work at the German Cancer Research Center and the EML Heidelberg, a small start‐up company, I moved to the University of Manchester before accepting a professorship at the University of Münster in 2003.

## 2 What Got You Interested in Biology? When Did You Know This Was Your Field?

I was always intrigued by how living systems manage to innovate and become—seemingly—more complex. Biology is full of examples where new functions arise from old parts—or sometimes from scratch. Coming from a theoretical and computational background, I often ended up sitting on a fence or in a nowhere land—sometimes too mathematical for biologists, at other times too biological for physicists or computer scientists. However, over time, this interdisciplinary space turned out to be where many of the most exciting questions arise. Taking such diverse perspectives turned out to be crucial for our discoveries and remains central to my lab's philosophy.

## 3 Which Achievement Are You Most Proud of?

I like that I have always been working on what interests me, but managed to move from one subject to another and often return to prior questions. For example, our recent research area on birth and evolution of protein‐coding de novo genes has been particularly rewarding as it emerged from a prior main research area on module protein evolution and fed into my oldest research area from the 1990s which aimed at understanding where in sequence space functional proteins are and how they can be evolutionary converted into each other. While new genes were long thought to evolve mostly via duplication, we have shown that entirely new protein‐coding genes can emerge from noncoding DNA, and often become functional and important (Schmitz et al. 2018; Vakirlis et al. 2020). In insects such as *Drosophila* and ants, these genes contribute to reproduction, development, and social behaviors. Earlier on, we studied protein domain evolution, showing how domain rearrangements drive functional innovation and how evolution innovates by reusing existing modules (Bornberg‐Bauer and Albà 2013).

## 4 What Inspired the GEvol Priority Programme, and What Do You Hope It Will Achieve?

The GEvol programme was inspired by the need to understand how genome‐level changes translate into evolutionary innovations. We now have unprecedented genomic data and methods to study gene birth, regulatory evolution, and novel molecular functions, but integrating these to explain phenotypic novelty remains a grand challenge. GEvol brings together evolutionary genomics, developmental biology, and computational science to address this. My hope is that it not only deepens our knowledge but also builds a vibrant, collaborative community advancing evolutionary innovation research.

## 5 What Is Your Favorite Paper?

Actually, a fairly old paper by David Lipman and Wilbur (1991), who were, to the best of my knowledge, the first to use simple models of biopolymers to explore how protein families distribute in sequence space. They demonstrated the paramount importance of neutral drift for reaching new innovative protein families.
